# Neuroprotection after a first episode of mania: a randomized controlled maintenance trial comparing the effects of lithium and quetiapine on grey and white matter volume

**DOI:** 10.1038/tp.2016.281

**Published:** 2017-01-24

**Authors:** M Berk, O Dandash, R Daglas, S M Cotton, K Allott, A Fornito, C Suo, P Klauser, B Liberg, L Henry, C Macneil, M Hasty, P McGorry, Cs Pantelis, M Yücel

**Affiliations:** 1 IMPACT Strategic Research Centre, School of Medicine, Deakin University, Geelong, VIC, Australia; 2 Orygen, The National Centre of Excellence in Youth Mental Health, Parkville, VIC, Australia; 3 Centre for Youth Mental Health, University of Melbourne, Parkville, VIC, Australia; 4 Orygen Youth Health Clinical Program, Parkville, VIC, Australia; 5 Barwon Health and the Geelong Clinic, Swanston Centre, Geelong, VIC, Australia; 6 Florey Institute for Neuroscience and Mental Health, Kenneth Myer Building, Royal Parade, Parkville, VIC, Australia; 7 Brain and Mental Health Laboratory, School of Psychological Sciences, Monash Institute of Cognitive and Clinical Neurosciences, Monash University, Clayton, VIC, Australia; 8 Department of Psychiatry, Melbourne Neuropsychiatry Centre, University of Melbourne and Melbourne Health, Carlton South, VIC, Australia; 9 Division of Medical Imaging and Technology, Department of Clinical Science, Intervention and Technology (CLINTEC), and Division of Psychiatry, Karolinska Institutet, Stockholm, Sweden

## Abstract

Lithium and quetiapine are effective treatments for bipolar disorder, but their potential neuroprotective effects in humans remain unclear. A single blinded equivalence randomized controlled maintenance trial was conducted in a prospective cohort of first-episode mania (FEM) patients (*n*=26) to longitudinally compare the putative protective effects of lithium and quetapine on grey and white matter volume. A healthy control sample was also collected (*n*=20). Using structural MRI scans, voxel-wise grey and white matter volumes at baseline and changes over time in response to treatment were investigated. Patients were assessed at three time points (baseline, 3 and 12-month follow-up), whereas healthy controls were assessed at two time points (baseline and 12-month follow-up). Patients were randomized to lithium (serum level 0.6 mmol l^−1^, *n*=20) or quetiapine (flexibly dosed up to 800 mg per day, *n*=19) monotherapy. At baseline, compared with healthy control subjects, patients with FEM showed reduced grey matter in the orbitofrontal cortex, anterior cingulate, inferior frontal gyrus and cerebellum. In addition, patients had reduced internal capsule white matter volume bilaterally (*t*_1,66_>3.20, *P*<0.01). Longitudinally, there was a significant treatment × time effect only in the white matter of the left internal capsule (F_2,112_=8.54, *P*<0.01). *Post hoc* testing showed that, compared with baseline, lithium was more effective than quetiapine in slowing the progression of white matter volume reduction after 12 months (*t*_1,24_=3.76, *P*<0.01). Our data support the role of lithium but not quetiapine therapy in limiting white matter reduction early in the illness course after FEM.

## Introduction

Many people with bipolar disorder (BD) manifest a progressive course. This observation has been repeatedly verified^1, 2^ and is manifested by progressively shorter inter-episode intervals, increasing rates of functional impairment, comorbidity, suicide, hospitalisation and reduced treatment responsiveness.^3, 4, 5, 6, 7, 8^ Moreover, there are replicated reports of changes over time in brain volume, suggesting that these neuroanatomical alterations might be more pronounced with repeated episodes. A larger number of episodes have been associated with decreased cortical and subcortical structures including prefrontal and temporal cortices, the hippocampus and the striatum^9, 10, 11, 12^ accompanied with an increase in lateral ventricular volume.^13, 14, 15^

The timing of these serial changes in clinical and imaging variables remains uncertain. What is probable is that people at risk who later go on to develop BD do not, on aggregate, demonstrate marked deficits. These appear with the onset of the disorder, and are evident in an established disease.^16^ Similarly, structural changes are inconsistent at a first episode, with some studies showing that individuals at a first episode manifest volumetric findings similar to controls,^17, 18^ whereas other studies suggest that changes are already evident.^19^ Either way, the first episode appears to be the soonest pragmatic point to commence therapeutic strategies with neuroprotective potential.

A considerable body of evidence from non-randomized studies suggests that lithium may be associated with preservation or increase in cortical grey matter^20, 21^ including the prefrontal cortex,^22^ amygdala and hippocampus.^23^ Evidence supporting a protective role for antipsychotics such as quetiapine largely draws on the rather uncertain schizophrenia literature. While some evidence points to a protective role for quetiapine that ceases after abstinence,^24^ other studies show either an increase only with typical antipsychotics,^25^ mixed findings of volumetric increases and reductions ^26^ or dose-dependent effects.^27^ A naturalistic study that examined longitudinal changes in grey and white matter in a cohort of schizophrenia and BD patients, treated with second-generation antipsychotics including quetiapine, found a significant reduction in the frontal cortex in the schizophrenia group and no change in the BD group.^17^

Despite the considerable evidence that atypical agents have clinical utility in BD, there is a lack of randomised controlled trials with imaging endpoints focusing on volumetric changes in response to treatment. To examine which agent may have better neuroprotective properties, a cohort of individuals were studied who had been stabilized after a first episode of mania on a combination of lithium and quetiapine and then randomized to either lithium or quetiapine monotherapy. Voxel-based morphometry was utilised to determine white and grey matter volume changes at baseline and at 3 and 12 months in treatment groups in comparison with healthy controls. We hypothesised that first-episode mania (FEM) patients would show altered brain volume compared with control subjects at baseline and that lithium and quetiapine treatments would be equivalent in affecting changes in brain volume in the first year following a first episode of mania.

## Materials and methods

### Study design

A single-blind controlled randomised parallel group design was conducted over 52 weeks at two sites in Melbourne, Australia; Orygen, The National Centre of Excellence in Youth Mental Health, the Early in Life Mental Health Service and the Recovery and Prevention of Psychosis services at Monash Health. In this trial, all individuals presenting with an acute first episode of mania with psychotic features were stabilised on a combination of quetiapine plus lithium in an open manner as part of a routine care protocol. Following the provision of informed consent, patients were randomised after remission (a period of between 2 and 3 months) to either lithium or quetiapine monotherapy. The study was conducted according to the Good Clinical Practice guidelines and was approved by all relevant ethical committees.

### Participants

Patients meeting Diagnostic and Statistical Manual of Mental Disorders, 4th Edition, Text Revision (DSM-IV-TR) criteria for a manic episode on the Structured Clinical Interview for DSM-IV-TR—Patient Edition (SCID-I/P)^28^ were recruited between December 2008 through December 2013. Individuals with a first-episode illness of bipolar I disorder, substance-induced mood disorder or schizoaffective disorder and aged 15–25 years were included. Other inclusion criteria included: a Young Mania Rating Scale (YMRS) score of at least 20 for the acute phase of a first episode of mania; no previously treated manic episode(s); capacity to provide informed consent to the study and comply with study procedures; be utilising effective contraception if female and to have been on quetiapine and lithium as standard therapy for at least 1 month before randomisation.

Exclusion criteria from the trial included: patients with a known or suspected clinically relevant systemic medical disorder; pregnant or lactating females; patients who had a prior sensitivity or allergy to quetiapine, lithium or their components; inability to comply with either the requirements of informed consent or the treatment protocol; non-fluency in English; history of epilepsy; clinically relevant biochemical or haematological abnormalities at baseline; patients at immediate risk of self-harm or risk to others; organic mental disease, including mental retardation (full-scale intelligence quotient <70); and an absolute neutrophil count of ⩽1.5 × 10^9^ l^−1^. Use of cytochrome P450 3A4 inhibitors in the 14 days preceding enrolment was not permitted including ketoconazole, itraconazole, fluconazole, erythromycin, clarithromycin, troleandomycin, indinavir, nelfinavir, ritonavir, fluvoxamine and saquinavir. Similarly, the use of cytochrome P450 inducers was not permitted in the 14 days preceding enrolment including phenytoin, carbamazepine, barbiturates, rifampacin, St John’s Wort or glucocorticoids. Extra exclusion criteria applied to individuals with diabetes mellitus (DM): unstable DM defined as enrolment glycosylated haemoglobin (HbA1c) >8.5; admission to hospital for treatment of DM or DM-related illness in the previous 12 weeks; not under physician care for DM; physician responsible for patient’s DM care did not indicate that patient’s DM was controlled; physician responsible for patient’s DM care did not approve patient’s participation in the study; had not been on the same dose of oral hypoglycaemic drug(s) for the 4 weeks before randomisation (8 weeks for thiazolidinediones); and daily insulin had been more than 10% outside their mean monthly dose on one or more occasions in the preceding 4 weeks.

All individuals presenting with an acute first manic episode with psychotic features were acutely stabilised on a combination of quetiapine plus lithium in an open manner as part of a routine care protocol. Individuals received quetiapine at a dose determined by the treating clinician. Optimal serum lithium levels targeted in the acute phase were between 0.8 and 1.0 mmol l^−1^. Following clinical stabilisation (based on the global impression of the treating clinician or team), subjects were randomised to either quetiapine or lithium. Lithium levels of 0.6–0.8 mmol l^−1^ were targeted in the maintenance phase, and the quetiapine dose was determined by the treating clinician (up to 800 mg per day).

### Randomisation and blinding

At the discretion of the treating team, an independent statistician generated a computerised randomisation sequence 2–3 months following stabilisation from a FEM. A randomisation log was established and a set of sequentially ordered envelopes was kept in a locked filing cabinet at the Orygen Research Centre site. Care was taken to maintain the single-blind nature of the study; patients and clinical staff including treating psychiatrist and case managers knew which treatment the patient was receiving while research staff including research assistants, neuropsychologists and all individuals involved in neuroimaging, analysis and data management remained blinded to this information. Participants were instructed not to communicate information regarding treatment to the research assistants and imaging personnel by the study clinicians, and no information regarding treatment was communicated at team meetings or was included in the study files to which the research assistants had access.

### Procedure

Clinical assessments were carried out at baseline and on fortnightly intervals for the first month, then on a monthly basis for the following two months and then at three monthly intervals thereafter concluding at the 12-month time point. The clinical assessment included observer-based ratings using the YMRS for manic symptoms, the Montgomery-Åsberg Depression Rating Scale^29^ for depressive symptoms; the Brief Psychiatric Rating Scale^30^ for overall psychopathology and severity of psychotic symptoms; and the Clinical Global Impression scale for use in BD^31^ to determine overall symptom severity. Control subjects underwent MRI scanning at baseline and at the 12-month time points, whereas FEM patients were scanned at baseline, 3 and 12-month time points to better assess response to treatment.

### MRI data acquisition

3 T Siemens Trio Tim scanner (32 channel head coil) at the Murdoch Children’s Research Institute in Melbourne, Australia, was used to acquire high-resolution structural T1 Magnetisation-Prepared RApid Gradient-Echo (MPRAGE^32^) scans for each subject. Image acquisition parameters at every time point were as follows: 192 sagittal slices with a nominal 1 mm^3^ voxel size, 256 mm × 232 mm field-of-view and a matrix size of 256 × 192 pixel resolution, 2000 ms repetition time and 2.24 ms echo time. Structural images of 25 patients were sampled at twice the resolution resulting in a matrix size of 512 × 384 pixels and an in-plane resolution of 0.5 mm × 0.5 mm.

### Voxel-based morphometry (pre-processing)

Pre-processing and post-processing for baseline and longitudinal comparisons were carried out in the VBM8 toolbox implemented in Statistical Parametric Mapping (SPM8; http://www.fil.ion.ucl.ac.uk/spm/software/spm8/) running in Matlab v.8.1 (MathWorks, Matick, MA, USA). Structural images were routinely inspected for artifacts and gross abnormalities. For baseline comparison voxel-wise analyses of brain gray matter volume and white matter volume differences were conducted using the Diffeomorphic Anatomical Registration Through Exponentiated Lie Algebra (DARTEL^33^). Briefly, each participant’s T1-weighted anatomical scan was segmented into distinct tissue compartments and spatially normalised. A study-specific template was generated by normalising each participant’s segmented grey or white matter image to a common space. Native-space grey or white matter images were then spatially normalised to this template. Modulation (nonlinear only) normalisation was used to preserve the amount of grey and white matter. The resulting modulated and spatially normalised segments were then smoothed with a 6.0 mm full width at half maximum spatial smoothing kernel in order to minimise anatomical residuals from registration. Grey and white matter segments were checked for segmentation and normalisation artifacts. Intracranial volume was calculated from the raw anatomical images in the subject’s native space as the total volume of grey matter, white matter and cerebrospinal fluid.

For longitudinal comparison, the VBM8 longitudinal pipeline was used. Briefly, for each subject, after follow-up images were initially realigned to baseline, a mean image was created and used as a reference image to which baseline and longitudinal images were realigned again. The realigned images at each time point were then corrected for signal inhomogeneity (bias correction) and segmented. Spatial normalisation using DARTEL was applied to the reference image, after which normalisation parameters were applied to the realigned and bias-corrected segments.

### Statistical analyses

Baseline differences in grey and white matter volume between FEM patients (collapsed across treatment groups) and control subjects were tested using the General Linear Model as implemented in the FSL feature Randomise (http://fsl.fmrib.ox.ac.uk/fsl/fslwiki/Randomise). All results were corrected for type I error with a nonparametric threshold-free cluster enhancement procedure employing 1000 permutations.^34^ Age and gender were included as covariates of no interest. Results were considered significant if they passed a threshold *P*<0.01. The results were then saved to mask treatment × time interaction results, thus ensuring that treatment effects were investigated in brain regions of primary pathophysiological importance to BD.

**Figure 1:**
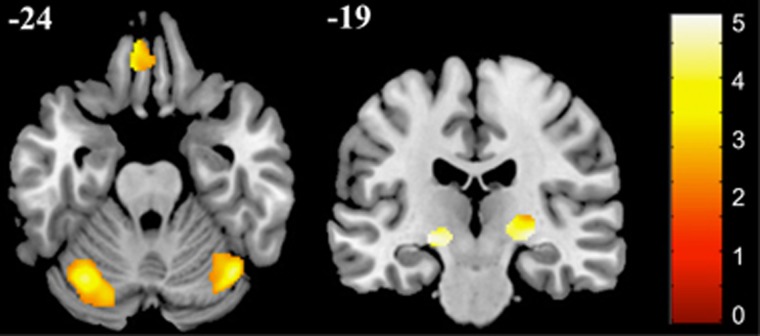
*Z*-score statistical map of reduced grey matter (left inset) and white matter (right inset) volume in first-episode mania patients (*N*=39) when compared with healthy control subjects (*N*=30) at baseline. Right hemisphere is shown on the right. Numbers represent MNI coordinates in each corresponding plane. Results are threshold-free cluster-enhancement-corrected for multiple comparison (*P*_TFCE_<0.01). See Table 2 for more information. MNI, Montreal Neurological Institute.

To examine changes in grey and white matter volume in response to treatment over time, preprocessed whole-brain grey and white matter segments were entered into separate flexible factorial models with group (control, quetiapine and lithium) and time (baseline, 3 months and 12 months) as factors and age, gender, DSM-IV-TR diagnosis (see Supplementary Table 1 for more information) and psychotropic medication as covariates of interest. In this analysis the effect of lithium versus quetiapine treatment on longitudinal changes in baseline differences in grey and white matter (treatment × time interaction) was the outcome measure. Group × time interactions were modelled using an F-test masked with the between-group difference results generated at baseline. Interaction results were corrected for multiple comparisons using small-volume correction as implemented in SPM8 with the between-group difference results mask generated at baseline as the volume of interest. Results that survived a threshold of *P*<0.01 were considered significant. A group × time interaction plot was generated by extracting the first eigenvariates (weighted averages) of white matter volume estimates from clusters that survived correction for multiple comparisons (Figure 1). Three months' follow-up data were compared separately for treatment groups exclusive of control data. Three months' follow-up data for control subjects were estimated by linear interpolation of baseline and 12 months data points and included for presentation purposes only.

## Results

Of the 61 recruited subjects, 11 in the quetiapine treatment group and 9 in the lithium treatment group were excluded at or before randomisation took place for the following reasons: relapse at baseline or before commencement of monotherapy (*n*=5); self-ceasing of all medications (*n*=3); non-stability on monotherapy (*n*=2); preference for the non-randomized medication (*n*=3); clinician withdrawal due to side effects (*n*=2); and treatment disengagement (*n*=4). Twenty-one patients were allocated to quetiapine treatment and 20 subjects were allocated to lithium. Two additional subjects were excluded at baseline; one subject because of never being on monotherapy and one subject because of non-compliance to randomized medication, rendering a final sample of 19 subjects in the quetiapine group and 20 subjects in the lithium group. Three patients in the quetiapine group and four in the lithium group discontinued after their baseline participation.

Treatment groups were matched for age, gender, handedness and intelligence quotient, but only matched for age and handedness with the control group (Table 1). Treatment groups (lithium and quetiapine) clinical scores did not differ across all clinical scales at baseline, indicating that stabilisation was successful (Table 1).

FEM patients (collapsed across treatment groups) demonstrated reduced regional grey and white matter volume compared with control subjects at baseline (*P*<0.01 corrected; Figure 1 and Table 2). Specifically, patients showed reduced grey matter volume in the orbitofrontal cortex, anterior cingulate cortex and the inferior frontal gyrus, as well as in the cerebellum (Figure 1 and Table 2). In addition, patients had reduced internal capsule white matter volume bilaterally (Figure 1 and Table 2). These findings clarify brain areas affected in participants stabilized in the aftermath of a FEM, and before monotherapy was commenced.

**Figure 2:**
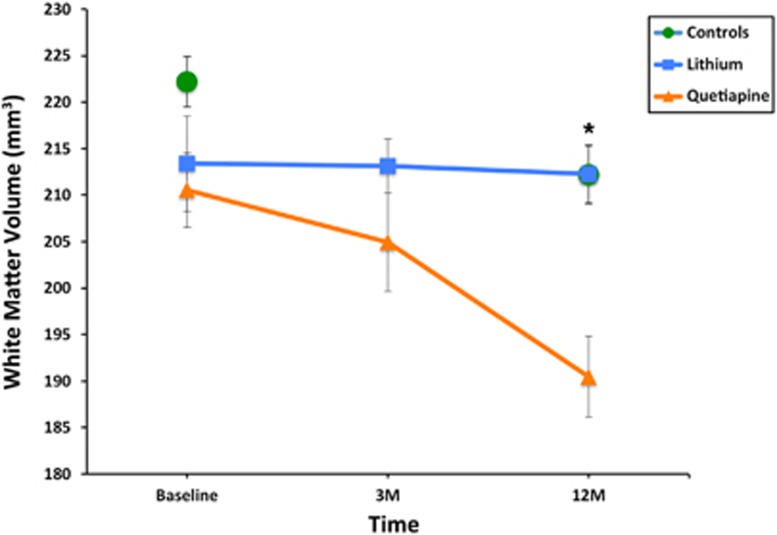
Time plot of significant changes in white matter volume over time in the left internal capsule (inset). Asterisk: significant between treatment group differences corrected (*P*_SMV_<0.01); error bars represent s.d. See Results section, Table 2 and Supplementary Materials for more information.

Longitudinally, there was a significant treatment × time effect only in the white matter of the left internal capsule (F_2,112_=10.44, *P*<0.01; Table 2 and Figure 2). To further assess whether the treatment effect exceeds the test–retest variability in control subjects, we assessed treatment × time interaction for each of the treatment group separately with the control group. We found no group × time interaction effect for the lithium group when compared with healthy control subjects and only in the quetiapine group (F=12.32, *P*<0.01 corrected). *Post hoc* testing showed that lithium was more effective than quetiapine in slowing the progression of white matter volume reduction as suggested by the difference score in white matter estimates between baseline and 12 months only (*t*_1,24_=3.76, *P*<0.01; Figure 2) but not after 3 months (*t*_1,27_=1.3, *P*=0.21). There were no other reductions or increases in grey or white matter volumes over time across any of the groups.

## Discussion

To the best of our knowledge, this is the first longitudinal structural neuroimaging study to characterize the neuroprotective effects of lithium and quetiapine in participants following a FEM. Compared with healthy controls, FEM patients demonstrated reduction in brain volume in a number of previously identified areas,^12, 35, 36^ including the orbitofrontal cortex, anterior cingulate cortex, inferior frontal gyrus and cerebellum at baseline. Volumetric reductions in white matter were restricted to the internal capsule bilaterally. Furthermore, we assessed differences in the effect of treatment across three time points, which has not previously been done. We found a significant effect of treatment on white matter, with the lithium group showing an attenuated reduction of white matter volume over time compared with the quetiapine group at 12 months of follow-up.

At baseline, we identified between-group differences confined to structures widely implicated in BD.^37^ We did not identify any regional changes in disease-specific grey matter across groups over time, and this absence of change is corroborated by another longitudinal study of FEM patients with BD.^38^ However, there are structural neuroimaging findings that suggest a progressive grey matter loss in the prefrontal cortex and anterior cingulate cortex in BD.^39^ The absence of grey matter alterations over time may be a result of a neuroprotective effect of medications or due to common neuropathology limited to psychotic illnesses that differentiates after first episode of mania.^38, 40^ This notion is supported by finding that progressive loss of grey matter in both the cingulate cortex and the cerebellum have been shown to be specific to subjects who later on developed psychosis.^41^

The superiority of lithium on white, but not grey, matter outcomes contrasts with previous findings.^12, 35^ Demonstrating an increased volume in the anterior cingulate cortex,^12^ hippocampus and amygdala^42^ was associated with lithium treatment in BD patients. Nonetheless, our finding of no change in grey matter over time is in agreement with a number of other studies that found either no change in grey matter volume^43^ or more extensive changes in white matter volume only.^38, 40^ The discrepancy in these findings can be attributed to the relatively lower incidence of multiple episodes and hence abnormalities severity in this sample compared with other studies^15^ and the relative age of participants.^44^

Although reduced white matter in the internal capsule volume was detected bilaterally at baseline, we detected a treatment effect only in the left hemisphere. This result may suggest that left white matter volume is more susceptible to illness processes in FEM patients in this brain region. This notion is in agreement with previous findings, showing a longitudinal reduction in white matter volume to be more affected in the left hemisphere (6.5%) in bipolar patients when compared with baseline.^45^ Furthermore, structural imaging studies investigating aspects of water diffusion in white matter fibre tracts have shown reduced fractional anisotropy in the left internal capsule only^46^ and loss of the normal left-sided symmetry in the posterior limb of the internal capsule,^47^ suggesting specific microstructural abnormality to the left hemisphere.

The duration of lithium treatment in this study is worth considering as it points to an effective cessation of white matter loss during the first 3 months and a subsequent normalisation of this effect to similar rate to that expected in control subjects (Figure 2). Previous studies demonstrated that among people with BD those with a greater duration of treatment with lithium manifested better adjusted white matter^48^ as well as grey matter integrity.^49^ In addition, there are preliminary reports of associations between white matter hyperintensities and better lithium response.^50^ These findings strongly suggest that lithium has a role in myelin physiology. In a crush injury model, for example, lithium is shown to enhance the re-myelination of peripheral nerves.^51^ An operative pathway whereby lithium acts on white matter seems to be via glycogen synthase kinase 3-β.^52^ The mechanism involves regulation of the genes for myelin basic protein and proteolipid protein expression in mouse oligodendrocytes.^53^

The results of this study do not support a neuroprotective role for quetiapine nor do they support a neurotoxic effect. This is clearly demonstrated by the fact that treatment groups did not differ on measures of both grey and white matter at baseline or in grey matter longitudinally. Rather, quetiapine seems to take much longer to exert an effect on white matter, suggesting perhaps a slower and less potent effect than lithium. Despite being a multisite study conducted over 5 years, the sample size of this study was limited. This is driven by the low incidence of FEM, together with the requirement to have been treated and stabilized on a combination of lithium and quetiapine, and to then agree to randomisation, be happy with the randomized agent and the trial assessments. As expected, there was a group of individuals who did not complete the trial, decreasing power at the final time point.

White matter neuroimaging metrics that reflect axonal structure, such as fractional anisotropy, and the mean and radial diffusivity are altered in people with BD compared with controls.^54^ Alterations in white matter microarchitecture have also been shown to be associated with poorer treatment response.^55^ This suggests that there is a process of disturbed myelination in the disorder that is seemingly not associated with axonal loss.^56^ Such changes can only be assessed with diffusion imaging that may prove more sensitive to axonal pathology than voxel-based morphometry analysis.

That most patients had psychotic symptoms at baseline means that these data cannot be extrapolated to non-psychotic individuals. The cohort included individuals with bipolar and schizoaffective disorder, although only a small number of participants had the latter diagnosis. Data regarding quetiapine cannot be extrapolated to other antipsychotics. To make the trial feasible, concomitant medications were allowed, and their role cannot be excluded. Dropout rates of around a third are not unexpected in a first-episode population, and the results need to be interpreted cognizant of this characteristic. Similarly, there was an imbalance between patients and controls regarding intelligence quotient and gender, which is a limitation. High rates of substance use in the cohort may have influenced the results, although both treatment groups were matched for subjects with substance/alcohol abuse and hence the results are not likely to be attributed to their effect. Because substance abuse is very common in this population, it was allowed to enhance generalisation. The randomized design, the two post-baseline scans using a single scanner and the 1-year follow-up are strengths of the design.

In summary, the data support the role of lithium in preventing white matter changes after a first episode of mania. In addition, given the progressive nature of the disorder, and the lack of data showing that established cognitive or imaging changes could be reversed, these data support the continuing use of lithium from the earliest stages of the disorder, pragmatically translated as the first episode. They thus challenge earlier guidelines, suggesting that one might wait for the passage of several episodes before commencing lithium.^57, 58, 59, 60, 61^

## Figures and Tables

**Table 1:** Demographic and clinical characteristics of FEM patients and healthy control subjects

*Demographics*	*Control* N=*30*		*Quetiapine (*N=*19)*		*Lithium (*N=*20)*		*Statistics*	
	N	*%*	N	*%*	N	*%*	χ^*2*^	P
*Gender*								
Male	12	40	14	70	16	84	9.87	0.007
Female	18	60	5	30	4	16		
								
*Handedness*^a^								
Right-handed	26	93	15	83	19	95	3.64	0.162
Left-handed	2	7	3	17	0	0		
								
*Age*	*Mean*	*s.d.*	*Mean*	*s.d.*	*Mean*	*s.d.*	*F*	
	21.40	2.46	21.47	2.14	21.45	2.31	0.145	0.88
Premorbid IQ (WTAR)	105.43	10.82	92.89	13.80	96.71	13.89	11.37	0.001

*Baseline clinical scores*^b^ *(total)*	*Control*		*Quetiapine*		*Lithium*		*Statistics*	
	*Mean*	*s.d.*	*Mean*	*s.d.*	*Mean*	*s.d.*	*Mann–Whitney* Z	P
YMRS	—	—	1.86	2.21	2.85	4.4	−0.074	0.941
MADRS	—	—	7.05	8.63	7.05	9.09	−0.667	0.505
CGI-BP (Severity of overall bipolar)	—	—	1.89	1.52	2.10	1.48	−0.677	0.498
CGI-BP (Severity of mania)	—	—	1.00	0.00	1.20	0.70	−1.396	0.163
CGI-BP (Severity of depression)	—	—	2.00	1.49	2.10	1.71	−0.047	0.962
BPRS	—	—	32.47	8.42	33.80	10.19	−0.297	0.767

Abbreviations: BPRS, Brief Psychiatric Rating Scale; CGI-BP, Clinical Global Impressions scale for use in bipolar disorder; FEM, first-episode mania; IQ, intelligence quotient; MADRS, Montgomery Asperger Depressive Rating Scale; WTAR, Wechsler Test of Adult Reading (UK-scaled score); YMRS, Young Mania Rating Scale.

a Data for two subjects in the control group and one subject in the quetiapine and the lithium group were missing

.

b Follow-up data for four and seven subjects were missing from the quetiapine and lithium group, respectively.

**Table 2:** Brain regions demonstrating baseline differences as well as group × time interaction effect in grey and white matter volume between first-episode mania patients and control subjects

*Baseline comparison*	*Hemisphere*	*Peak MNI coordinates (*x,y,z*)*	*Cluster size voxels (mm*^*3*^)	z*-score*
*Grey Matter*				
Orbitofrontal cortex/gyrus rectus	Right	−3, 38, −27	170 (574)	4.56
Cerebellum	Left	−30, −66, −21	1190 (4016)	5.12
Cerebellum	Right	39, −63, −22	1755 (5923)	5.07
				
*White matter*				
Internal capsule	Right	20, −18, 2	336 (1134)	5.29
Internal capsule	Left	−22, -19, -6	171 (577)	5.37
				
*Group* × *time interaction (white matter)*				
Internal capsule	Left	−22, −19, −8	82 (277)	3.64

Abbreviation: MNI, Montreal Neurological Institute.

Results are corrected for multiple comparison (*P*<0.01).
