# Neuroprotection after a first episode of mania: a randomized controlled maintenance trial comparing the effects of lithium and quetiapine on grey and white matter volume

**DOI:** 10.1038/tp.2017.13

**Published:** 2017-02-21

**Authors:** M Berk, O Dandash, R Daglas, S M Cotton, K Allott, A Fornito, C Suo, P Klauser, B Liberg, L Henry, C Macneil, M Hasty, P McGorry, Cs Pantelis, M Yücel

**Correction to**: *Translational Psychiatry* (2017) **7**, e1011; doi:10.1038/tp.2016.281; published online 24 January 2017

The 14th author's name was presented incorrectly. The correct listing is C Pantelis.

