# Supplementary material for: Neuroprotection after a first episode of mania: a randomized controlled maintenance trial comparing the effects of lithium and quetiapine on grey and white matter volume
Source: Transl Psychiatry. 2017 Jan 24;7(1):e1011–. doi: 10.1038/tp.2016.281 (PMC5545739; doi:10.1038/tp.2016.281)
Supplement: Supplementary Materials [file tp2016281x1.doc]

**Supplementary Table 1**: DSM-IV-TR Axis I and Axis II diagnoses of FEM patients.

| **DSM Axis-I Clinical Diagnosis** | **Quetiapine Treatment (N=19)** | **Lithium**  **Treatment (N=20)** | **DSM Axis-II Clinical Diagnosis** | **Quetiapine Treatment (N=19)** | **Lithium**  **Treatment (N=20)** |
| --- | --- | --- | --- | --- | --- |
| Bipolar I Disorder | 17 | 16 | Cannabis Dependence/Abuse | 5 | 7 |
| Schizoaffective Disorder | 1 | 1 | Alcohol Dependence/Abuse | 5 | 5 |

**Supplementary Table 2**: A group x time interaction effect in left internal capsule white matter volume in first-episode mania patients after controlling for DSM-IV-TR Axis I and Axis II diagnoses and psychotropic medication in addition to age and gender. MNI: Montreal Neurological Institute. Results are viewed at (*P*<.05 uncorrected).

| **Group X Time Interaction**  **(White Matter)** | **Hemisphere** | **Peak MNI Coordinates**  **(x,y,z)** | **Cluster Size Voxels (mm^3^)** | **z-score** |
| --- | --- | --- | --- | --- |
| Internal Capsule | Left | -15, -22, -5 | 60 (202) | 2.76 |

**Supplementary Figure Legends**

**Supplementary Figure 1:** Box-plot representing white matter volume changes in the left internal capsule in healthy control subjects and FEM patients over time.

**Supplementary Figure 2:** F-test statistical map of group x time interaction effect in left internal capsule white matter volume in first-episode mania patients after controlling for DSM-IV-TR Axis I & II diagnoses and psychotropic medication in addition to age and gender. Right hemisphere is shown on the right. Results are viewed at (*P*<.05 uncorrected). See Supplementary Table 2 for more information.
