# Supplementary figures and images for: Neuroprotection after a first episode of mania: a randomized controlled maintenance trial comparing the effects of lithium and quetiapine on grey and white matter volume
Source: Transl Psychiatry. 2017 Jan 24;7(1):e1011–. doi: 10.1038/tp.2016.281 (PMC5545739; doi:10.1038/tp.2016.281)

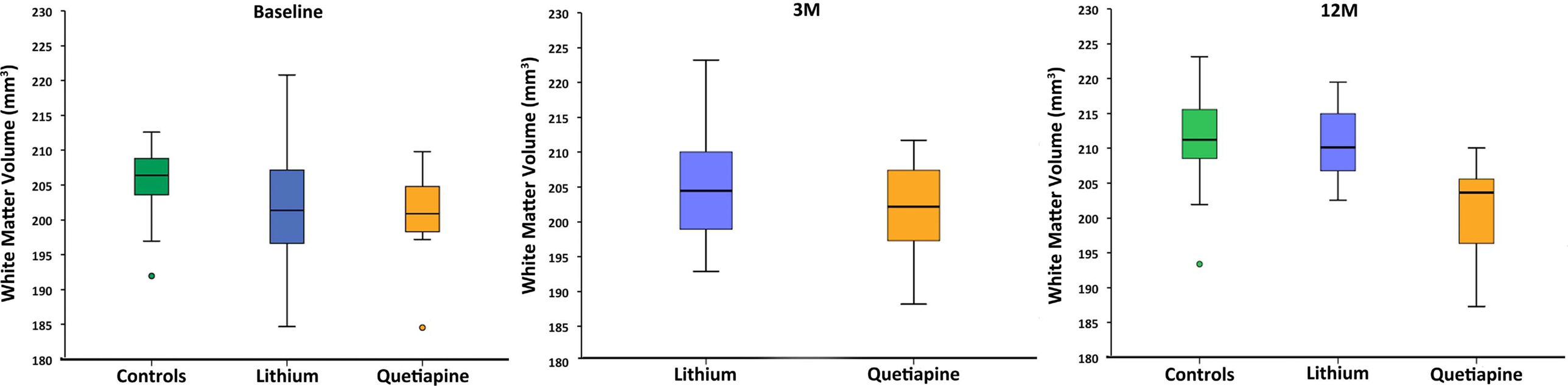

Supplement: Supplementary Figure 1 [file tp2016281x2.tif]

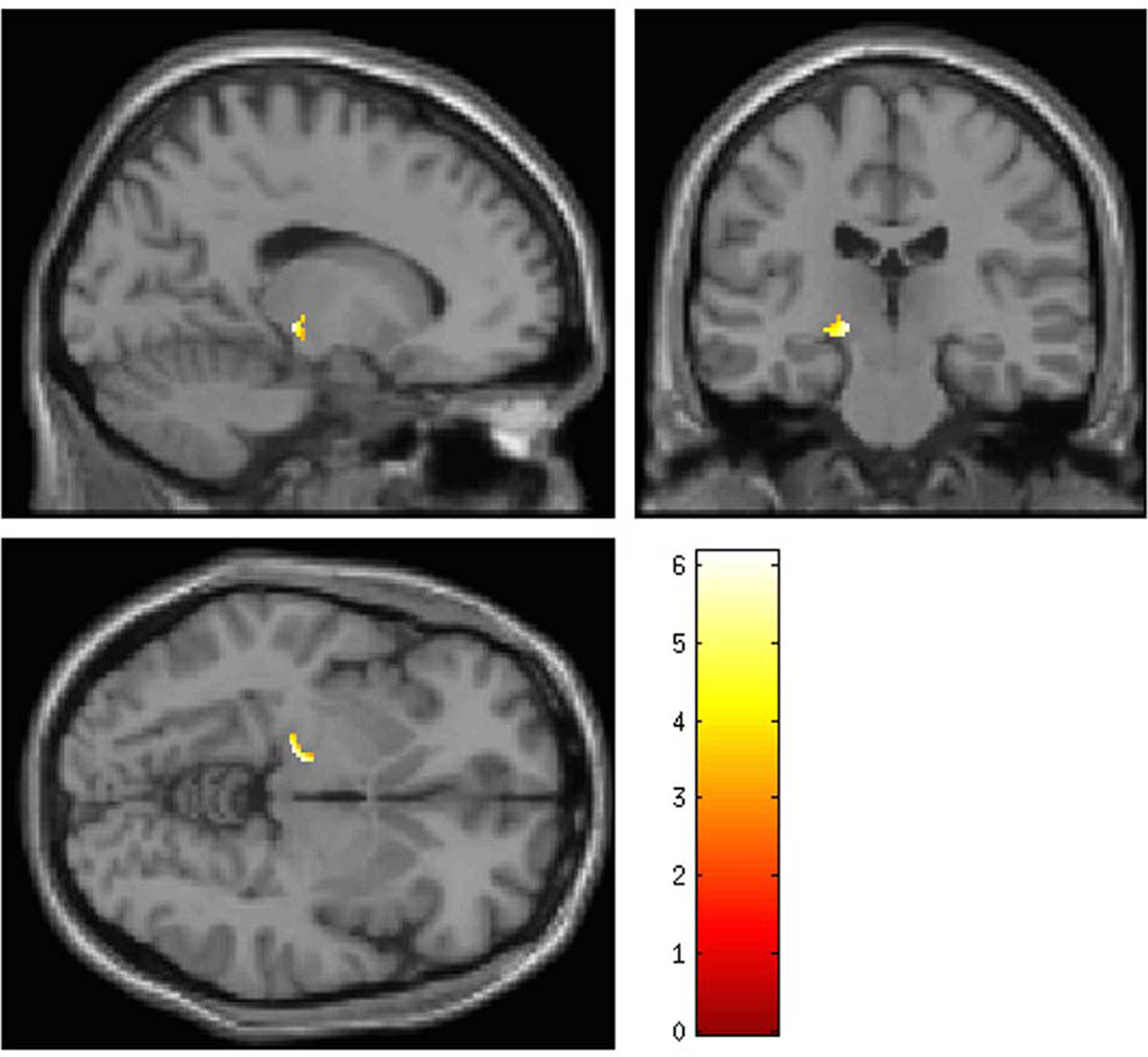

Supplement: Supplementary Figure 2 [file tp2016281x3.tif]
